# Novel gene arrangement in the mitochondrial genome of *Aspersentis megarhynchus* (Acanthocephala, Echinorhynchida, Heteracanthocephalidae), and its phylogenetic implications[Fn FN1]

**DOI:** 10.1051/parasite/2024064

**Published:** 2024-10-08

**Authors:** Yuan-Yuan Xie, Hui-Xia Chen, Tetiana A. Kuzmina, Olga Lisitsyna, Liang Li

**Affiliations:** 1 Hebei Collaborative Innovation Center for Eco‐Environment; Hebei Key Laboratory of Animal Physiology, Biochemistry and Molecular Biology; College of Life Sciences, Hebei Normal University 050024 Shijiazhuang Hebei Province P.R. China; 2 Hebei Research Center of the Basic Discipline Cell Biology; Ministry of Education Key Laboratory of Molecular and Cellular Biology 050024 Shijiazhuang Hebei Province P.R. China; 3 I. I. Schmalhausen Institute of Zoology National Academy of Sciences of Ukraine 15, Bogdan Khmelnytsky Street Kyiv 01030 Ukraine; 4 Institute of Parasitology, Slovak Academy of Sciences Hlinkova 3 Kosice 04001 Slovakia

**Keywords:** Acanthocephala, Heteracanthocephalidae, Nototheniidae, Mitochondrial genome, Phylogeny

## Abstract

The Heteracanthocephalidae Petrochenko, 1956 is a rare family of acanthocephalans mainly parasitic in fishes. The pattern of mitogenomic evolution of the Heteracanthocephalidae is still unknown, and the phylogenetic relationships of the Heteracanthocephalidae with the other 14 families within the order Echinorhynchida remain unclear. In the present study, the complete mitochondrial genome of *Aspersentis megarhynchus* (von Linstow, 1892) Golvan, 1960 was sequenced and annotated for the first time, which represents the first mitogenomic data for the genus *Aspersentis* and also for the family Heteracanthocephalidae. The mitogenome of *A. megarhynchus* has 14,661 bp and includes 36 genes, containing 12 protein-coding genes (PCGs) (missing *atp8*), 22 tRNA genes, and 2 ribosomal RNAs (*rrnS* and *rrnL*), plus two non-coding regions. Comparative mitochondrial genomic analysis revealed that the presence of translocations of several tRNA genes (*trnV*, *trnE*, and *trnT*) and the gene arrangement in the mitogenome of *A. megarhynchus* represents a new type in Acanthocephala. Moreover, the mitogenomic phylogenetic results based on concatenated amino acid sequences of 12 protein-coding genes strongly supported the validity of the Heteracanthocephalidae and suggested close affinity between the Heteracanthocephalidae and Echinorhynchidae in the order Echinorhynchida.

## Abstract (fr)

Les Heteracanthocephalidae Petrochenko, 1956 sont une famille rare d’acanthocéphales principalement parasites de poissons. Le schéma d’évolution mitogénomique des Heteracanthocephalidae est encore inconnu, et les relations phylogénétiques des Heteracanthocephalidae avec les 14 autres familles de l’ordre Echinorhynchida restent floues. Dans la présente étude, le génome mitochondrial complet d’*Aspersentis megarhynchus* (von Linstow, 1892) Golvan, 1960 a été séquencé et annoté pour la première fois, ce qui représente les premières données mitogénomiques pour le genre *Aspersentis* et également pour la famille Heteracanthocephalidae. Le mitogénome d’*A. megarhynchus* compte 14 661 pb et comprend 36 gènes, contenant 12 gènes codant pour des protéines (*atp8* manquant), 22 gènes d’ARNt et 2 ARN ribosomiques (*rrnS* et *rrnL*), plus deux régions non codantes. L’analyse génomique mitochondriale comparative a révélé que la présence de translocations de plusieurs gènes d’ARNt (*trnV*, *trnE* et *trnT*) et la disposition des gènes dans le mitogénome d’*A. megarhynchus* représentent un nouveau type chez les Acanthocephala. De plus, les résultats phylogénétiques mitogénomiques basés sur des séquences concaténées d’acides aminés de 12 gènes codant pour des protéines soutiennent fortement la validité des Heteracanthocephalidae et suggèrent une affinité étroite entre les Heteracanthocephalidae et les Echinorhynchidae dans l’ordre des Echinorhynchida.

## Introduction

The phylum Acanthocephala, commonly known as spiny- or thorny-headed worms, is an important group of zooparasites of veterinary, medical, and economic importance [45, 48, 49, 61]. It includes over 1,300 species assigned into 4 classes (Archiacanthocephala, Eoacanthocephala, Polyacanthocephala, and Palaeacanthocephala) and 10 orders [3, 25, 64]. Among them, Echinorhynchida Southwell & Macfie, 1925 is the largest order in Acanthocephala with 470 species occurring in teleost fishes, amphibians, and reptiles globally, which is divided into 15 families [2, 3, 6, 13, 25, 50, 55]. However, the evolutionary relationships of these 15 families remain unclear, due to the paucity and inaccessibility of genetic data.

The family Heteracanthocephalidae Petrochenko, 1956 is a rare group of acanthocephalans mainly parasitic in fishes [4, 31, 49, 51, 54]. It was erected by Petrochenko [49] and currently includes only three genera, namely *Aspersentis* Van Cleave, 1929 (type genus), *Bullockrhynchus* Chandra, Hanumantha-Rao & Shyamasundari, 1985 and *Sachalinorhynchus* Krotov & Petrochenko in Petrochenko, 1956 [3]. However, the systematic status of the Heteracanthocephalidae and the genus *Aspersentis* has been under debate for a long time [3, 4, 51]. Van Cleave [59] established the genus *Aspersentis* and assigned it to the family Rhadinorhynchidae Lühe, 1912. Later, Petrochenko [49] transferred *Aspersentis* into the family Arhythmacanthidae Yamaguti, 1935. Golvan [21] erected the family Aspersentidae Golvan, 1960 for the genus *Aspersentis*, which was subsequently reduced to a subfamily Aspersentinae Golvan, 1960 in the Heteracanthocephalidae [1, 3, 22].

Some previous studies proved that the nuclear and mitochondrial sequence data, including mitochondrial genomes, play important roles in the integrated taxonomy, population genetics, and phylogenetics of acanthocephalans [7, 9, 10, 18, 19, 33–35, 39–44, 56, 63]. However, GenBank contains limited sequence data from only two representatives of the Heteracanthocephalidae, *A. megarhynchus* (18S) and *Aspersentis* sp. (18S, 28S, and *cox1*). There are no data on the mitochondrial genome of heteracanthocephalid species reported so far.

In the present study, in order to enrich the genetic data and reveal the characterization of the complete mitochondrial genome of the Heteracanthocephalidae, the nuclear ribosomal DNA [including small ribosomal subunit (18S), large ribosomal subunit (28S), and internal transcribed spacer (ITS) sequences] of *A. megarhynchus* was sequenced, and the mitogenome of *A. megarhynchus* was annotated for the first time. Moreover, to evaluate the validity of the Heteracanthocephalidae and clarify the phylogenetic relationships among the families within the order Echinorhynchida, phylogenetic analyses based on concatenated amino acid sequences of 12 protein-coding genes (PCGs) of mitochondrial genomes were performed using maximum likelihood (ML) and Bayesian inference (BI), respectively.

## Material and methods

### Ethics and permits

The fish sampling was carried out in accordance with the National permit (series AP No. 046-14 from 11-2-2014) issued under the provisions of the Protocol on Environmental Protection to the Antarctic Treaty.

### Parasite collection and species identification

**Figure 1:**
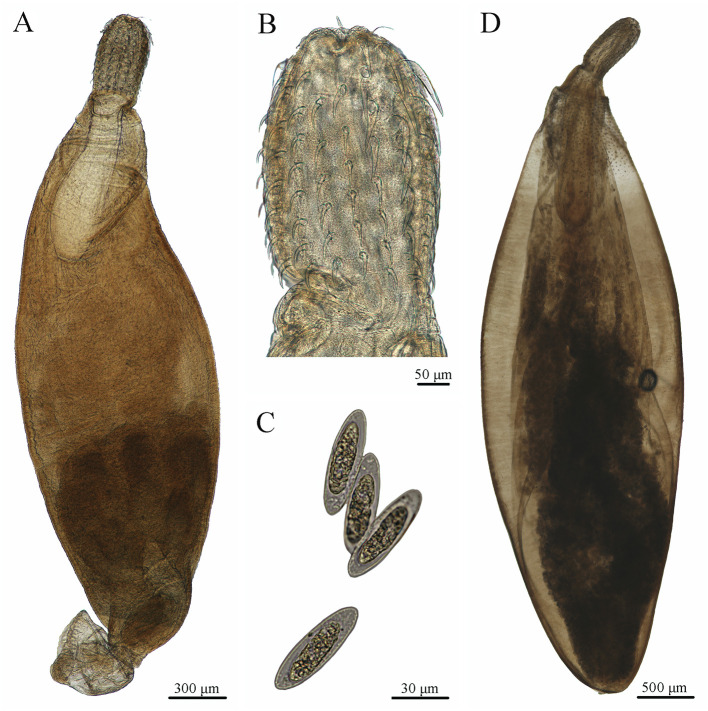
Photomicrographs of *Aspersentis megarhynchus* collected from *Notothenia coriiceps*. A: mature male; B: proboscis; C: eggs; D: mature female.

Acanthocephalans were isolated from the intestine of Antarctic black rockcod *Notothenia coriiceps* Richardson (Perciformes: Nototheniidae) off Galindez Island, Argentine Islands, West Antarctica (65°15′S, 64°16′W) during the 19th Ukrainian Antarctic expedition to the Ukrainian Antarctic Station (UAS) “Akademik Vernadsky” in April 2014–February 2015. Specimens were collected from fish intestines and stored in 70% ethanol for further study. The specimens were identified as *A. megarhynchus* based on morphological characters (Fig. 1), according to the previous studies [4, 30]. Voucher specimens of *A. megarhynchus* were deposited at the I. I. Schmalhausen Institute of Zoology, National Academy of Sciences of Ukraine, Kyiv, Ukraine (NC-49-2014-Acanth; NC-63-2014-Acanth; NC-72-2014-Acanth) and the College of Life Sciences, Hebei Normal University, Hebei Province, China (HBNU-A-F20240720CL).

### Molecular procedures

For molecular analysis, the total genomic DNA of *A. megarhynchus* was extracted using a Magnetic Universal Genomic DNA Kit (DP705) [Sangon Biotech (Shanghai) Co., Ltd., Shanghai, China], following the manufacturer’s instructions. The partial 18S region was amplified by polymerase chain reaction (PCR) using the forward primer (5′–AGATTAAGCCATGCATGCGTAA–3′) and the reverse primer (5′–TGATCCTTCTGCAGGTTCACCTAC–3′) [15]. The partial 28S region was amplified by PCR using four overlapping PCR fragments of 700–800 bp. Primers for 28S amplicon 1 were forward 5′–CAAGTACCGTGAGGGAAAGTTGC–3′ and reverse 5′–CAGCTATCCTGAGGGAAAC–3′; amplicon 2, forward 5′–ACCCGAAAGATGGTGAACTATG–3′ and reverse 5′–CTTCTCCAAC(T/G)TCAGTCTTCAA–3′; amplicon 3, forward 5′–CTAAGGAGTGTGTAACAACTCACC–3′ and reverse 5′–AATGACGAGGCATTTGGCTACCTT–3′; amplicon 4, forward 5′–GATCCGTAACTTCGGGAAAAGGAT–3′ and reverse 5′–CTTCGCAATGATAGGAAGAGCC–3′ [12]. The partial ITS region was amplified by PCR using the forward primer (5′–GTCGTAACAAGGTTTCCGTA–3′) and the reverse primer (5′–TATGCTTAAATTCAGCGGGT–3′) [29]. The cycling conditions were as described previously [35]. PCR products were checked on GoldView-stained 1.5% agarose gels and purified with a Column PCR Product Purification Kit (Sangon, Shanghai, China). Sequencing for each amplification product was carried out from both directions. Sequences were aligned using ClustalW2 and adjusted manually. The DNA sequences obtained herein were compared (using the algorithm BLASTn) with that available in the National Center for Biotechnology Information (NCBI) database (https://www.ncbi.nlm.nih.gov).

### Mitochondrial genome sequencing, assembly, and annotation

Total genomic DNA was sent to Novogene (Tianjin, China), where the library was prepared according to an internal protocol, before being sequenced on an Illumina NovaSeq 6000 platform. A total of 50 GB of clean 150 bp paired-end reads were obtained. GetOrganelle v1.7.2a [26] was used to assemble the mitochondrial genome. The mitochondrial genome was roughly annotated for the protein-coding genes (PCGs), transfer RNA (tRNA), and ribosomal RNA (rRNA) using the MitoS web server (http://mitos.bioinf.uni-leipzig.de/index.py) and MitoZ v2.4 [36]. The open reading frame (ORF) of each PCG was manually confirmed based on the invertebrate mitochondrial genetic code using the ORF finder (https://www.ncbi.nlm.nih.gov/orffinder/). Some tRNA genes unidentified by MitoS or MitoZ were discovered through BLAST, based on a database of the existing tRNA sequences of Acanthocephala. The secondary structures of tRNAs were predicted by the ViennaRNA module [23], building on MitoS2 [5] and RNA structure v6.3 [52], and manually corrected. The CGView online server V1.0 (http://stothard.afns.ualberta.ca/cgview_server/) was employed to visualize and depict gene element features. Base composition, amino acid usage, and relative synonymous codon usage (RSCU) were calculated by using a Python script (Supplementary file 1) referencing the codon adaptation index (CAI) [8]. Strand asymmetry was calculated using the formulae: AT skew = (A − T)/(A + T); GC skew = (G − C)/(G + C). The complete mitochondrial genome of *A. megarhynchus* obtained in this study has been deposited in the GenBank database (https://www.ncbi.nlm.nih.gov) under the accession number PP965112.

### Phylogenetic analyses

Phylogenetic analyses were conducted based on concatenated amino acid (AA) sequences of the 12 PCGs using maximum likelihood (ML) and Bayesian inference (BI). *Rotaria rotatoria* and *Philodina citrina* (Rotifera: Bdelloidea) were chosen as the out-group. The in-group included the newly sequenced *A. megarhynchus* and the other 39 species of acanthocephalans with mitogenomic data. Detailed information on the representatives of Acanthocephala included in the present phylogeny is provided in Table 1. The extracted amino acid sequences were aligned separately using MAFFT v7.313 under the iterative refinement method of E-INS-I [28]. The aligned AAs sequences were concatenated into a single alignment matrix by PhyloSuite v1.2.2 [62]. Substitution models were compared and selected according to the Bayesian Information Criterion (BIC) by using ModelFinder [27]. VT + F + I + G4 was identified as the best-fit substitution model. The Bayesian Information Criterion analysis was generated using MrBayes v3.2, with running for sampling tree topologies every 1,000 generations. The Markov Chain Monte Carlo process used randomly starting trees and involved four chains each in two parallel runs for 500,000 generations, with the first 25% of trees discarded as burn-in. The standard deviation of the split frequency value is lower than 0.01.

**Table 1:** Detailed information on representatives of Acanthocephala included in the present phylogeny.

Taxon	Order	Family	Species	Accession	Size (bp)	AT%	References
Outgroup							
Rotifera	Bdelloidea	Philodinidae	*Rotaria rotatoria*	NC_013568	15,319	73.2	[37]
			*Philodina citrina*	FR856884	14,003	77.7	[60]
Ingroup							
Archiacanthocephala	Moniliformida	Moniliformidae	*Moniliformis* sp.	OK415026	14,066	66.2	[9]
			*Moniliformis tupaia*	OP413683	14,150	63.8	Unpublished
	Oligacanthorhynchida	Oligacanthorhynchidae	*Macracanthorhynchus hirudinaceus*	NC_019808	14,282	65.2	[60]
			*Oncicola luehei*	NC_016754	14,281	60.2	[16]
Eoacanthocephala	Gyracanthocephala	Quadrigyridae	*Acanthogyrus cheni*	KX108947	13,695	65.3	[56]
			*Acanthogyrus bilaspurensis*	MT476589	13,360	59.3	[44]
			*Pallisentis celatus*	NC_022921	13,855	61.5	[47]
	Neoechinorhynchida	Neoechinorhynchidae	*Neoechinorhynchus violentum*	KC415004	13,393	59.4	[46]
			*Neoechinorhynchus qinghaiensis*	MW851291	13,271	65.8	Unpublished
			*Acanthocephalus* sp. (*Neoechinorhynchus* sp.)	MT345686	13,269	65.1	Unpublished
		Tenuisentidae	*Paratenuisentis ambiguus*	NC_019807	13,574	66.9	[60]
Palaeacanthocephala	Echinorhynchida	Arhythmacanthidae	*Heterosentis pseudobagri*	OP278658	13,742	62.5	[11]
		Cavisomidae	*Cavisoma magnum*	MN562586	13,594	63.0	[41]
		Echinorhynchidae	*Echinorhynchus truttae*	NC_019805	13,659	63.1	[60]
		Heteracanthocephalidae	*Aspersentis megarhynchus*	PP965112	14,661	64.6	Present study
		llliosentidae	*Brentisentis yangtzensis*	MK651258	13,864	68.3	[57]
		Pomphorhynchidae	*Longicollum* sp.	OR215045	14,632	55.8	Unpublished
			*Pomphorhynchus bulbocolli*	JQ824371	13,915	59.9	Unpublished
			*Pomphorhynchus laevis*	MN562482	13,881	57.5	Unpublished
			*Pomphorhynchus rocci*	JQ824373	13,845	60.7	Unpublished
			*Pomphorhynchus tereticollis*	JQ809452	13,890	56.8	Unpublished
			*Pomphorhynchus zhoushanensis*	MN602447	14,632	55.8	Unpublished
		Pseudoacanthocephalidae	*Pseudoacanthocephalus bufonis*	MZ958236	14,056	58.4	[63]
			*Pseudoacanthocephalus* sp.	OQ588705	14,883	61.5	Unpublished
		Rhadinorhynchidae	*Micracanthorhynchina dakusuiensis*	OP131911	16,309	56.8	[10]
			*Leptorhynchoides thecatus*	NC_006892	13,888	71.4	[58]
	Polymorphida	Centrorhynchidae	*Centrorhynchus clitorideus*	MT113355	15,884	55.5	[43]
			*Centrorhynchus milvus*	MK922344	14,314	54.5	[40]
			*Centrorhynchus aluconis*	KT592357	15,144	54.5	[18]
			*Sphaerirostris lanceoides*	MT476588	13,478	58.0	[42]
			*Sphaerirostris picae*	MK471355	15,170	58.1	[39]
		Polymorphidae	*Polymorphus minutus*	MN646175	14,149	64.4	[53]
			*Southwellina hispida*	NC_026516	14,742	63.9	[17]
			*Bolbosoma balaenae*	MZ357084	14,301	62.6	[14]
			*Bolbosoma capitatum*	MZ357085	14,319	63.9	[14]
			*Bolbosoma vasculosum*	MZ357087	14,313	63.9	[14]
			*Bolbosoma nipponicum*	OR468096	14,296	60.9	[33]
			*Corynosoma villosum*	OR468095	14,241	61.0	[33]
		Plagiorhynchidae	*Plagiorhynchus transversus*	NC_029767	15,477	61.1	[18]
Polyacanthocephala	Polyacanthorhynchida	Polyacanthorhynchidae	*Polyacanthorhynchus caballeroi*	NC_029766	13,956	56.3	[18]

The maximum likelihood (ML) inference was executed in IQTREE v2.1.2. Substitution models were compared and selected according to the Akaike Information Criterion (AIC) using ModelFinder. mtInv+F+R6 was identified as the best-fit substitution model. Nodal support for the ML tree was assessed using 1,000 bootstrap pseudoreplicates with ultrafast bootstrap approximation [24], while the other parameters were kept at their default values [20, 38]. The phylogenetic trees were visualized in iTOL v6.1.1 [32].

## Results

### Molecular characterization of nuclear ribosomal DNA of *Aspersentis megarhynchus*

#### Partial 18S region

Two 18S sequences of *A. megarhynchus* obtained herein had a length of 1,665 bp and were identical. GenBank contains 18S sequence data for two representatives of *Aspersentis*/Heteracanthocephalidae, namely *A. megarhynchus* (MW916820, MW916821) and *Aspersentis* sp. (OQ942219). Pairwise comparison of the 18S sequences of *A. megarhynchus* obtained herein with that of *Aspersentis* spp. showed no nucleotide variation (*A. megarhynchus*, MW916821) to 0.83% (*Aspersentis* sp., OQ942219) nucleotide divergence. The 18S sequences of *A. megarhynchus* obtained herein were deposited in GenBank (https://www.ncbi.nlm.nih.gov) (under accession numbers PP956812, PP956813).

#### Partial 28S region

Two 28S sequences of *A. megarhynchus* obtained herein are both 2,698 bp in length, with no nucleotide divergence detected. GenBank contains 28S sequence data for only one representative of *Aspersentis*/Heteracanthocephalidae: *Aspersentis* sp. (OQ947383). A pairwise comparison of the 28S sequences of *A. megarhynchus* obtained herein with that of *Aspersentis* sp. showed 7.38% nucleotide divergence. The 28S sequences of *A. megarhynchus* obtained herein were deposited in GenBank (https://www.ncbi.nlm.nih.gov) (under accession numbers PP958581, PP958582).

#### Partial ITS region

Two ITS sequences of *A. megarhynchus* obtained herein are both 533 bp in length, with no nucleotide divergence detected. In *Aspersentis*/Heteracanthocephalidae, there is no species with ITS sequences available in GenBank. The ITS sequences of *A. megarhynchus* obtained herein were deposited in GenBank (https://www.ncbi.nlm.nih.gov) (under accession numbers PP971137, PP971138).

### General characterization of the complete mitogenome of *Aspersentis megarhynchus*

**Figure 2:**
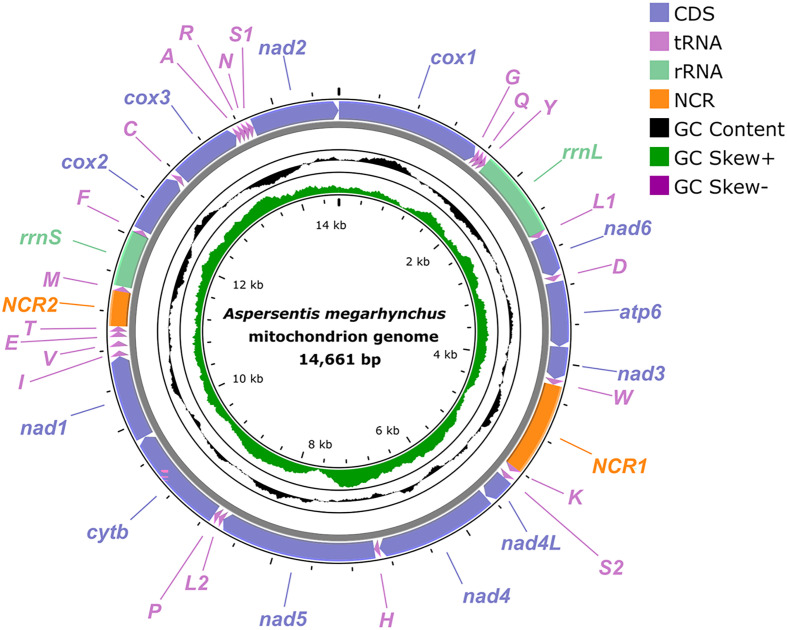
Gene map of the mitochondrial genome of *Aspersentis megarhynchus*. All 22 tRNA genes are nominated by the one-letter code with numbers differentiating each of the two tRNAs, serine and leucine. All genes are transcribed in the clockwise direction on the same strand. The outermost circle shows the GC content and the innermost circle shows the GC skew.

The complete mitogenome of *A. megarhynchus* is 14,661 bp in length and includes 36 genes, containing 12 PCGs (*cox1-3*, *nad1-6*, *nad4L*, *cytb* and *atp6*; missing *atp8*), 22 tRNA genes and 2 ribosomal RNAs (*rrnS* and *rrnL*), plus two non-coding regions (*NCR1* is 967 bp, located between *trnW* and *trnK*; *NCR2* is 366 bp, located between *trnT* and *trnM*) (Fig. 2, Table 2). All mitochondrial genes are encoded on the same strand and in the same direction. The overall A + T content in the mitogenome of *A. megarhynchus* is 64.6%, displaying a strong A + T bias. The nucleotide content of the mitogenome of *A. megarhynchus* is provided in Table 3.

**Table 2:** Organization of the mitochondrial genome of *Aspersentis megarhynchus*. “Ini/Ter cod” and “Int seq” indicates initial/terminal codons and the length of intergenic sequences, respectively.

Gene/Region	Position 5′ to 3′	Size (bp)	Ini/Ter cod	Anticodon	Int seq
*cox1*	1–1539	1539	GTG/TAA		−2
*trnG*	1538–1590	53		ucc	−13
*trnQ*	1578–1644	67		uug	−14
*trnY*	1631–1682	52		gua	0
*rrnL*	1683–2594	912			0
*trnL1*	2595–2648	54		uag	0
*nad6*	2649–3075	427	ATG/T		6
*trnD*	3082–3134	53		guc	11
*atp6*	3146–3829	684	ATA/TAA		0
*nad3*	3830–4175	346	GTG/T		0
*trnW*	4176–4237	62		uca	0
*NCR1*	4238–5204	967			0
*trnK*	5205–5265	61		uuu	38
*trnS2*	5304–5358	55		uga	−1
*nad4L*	5358–5633	276	GTG/TAG		8
*nad4*	5642–6902	1261	ATT/T		3
*trnH*	6906–6957	52		gug	0
*nad5*	6958–8619	1662	GTG/TAG		−1
*trnL2*	8619–8671	53		uaa	4
*trnP*	8676–8728	53		ugg	0
*cytb*	8729–9835	1107	GTG/TAA		3
*nad1*	9839–10733	895	ATG/T		0
*trnI*	10734–10793	60		gau	42
*trnV*	10836–10896	61		uac	43
*trnE*	10940–10992	53		uuc	1
*trnT*	10994–11049	56		ugu	0
*NCR2*	11050–11415	366			0
*trnM*	11416–11472	57		cau	0
*rrnS*	11473–12047	575			0
*trnF*	12048–12102	55		gaa	0
*cox2*	12103–12744	642	GTG/TAG		6
*trnC*	12751–12801	51		gca	8
*cox3*	12810–13535	726	ATG/TAA		−2
*trnA*	13534–13588	55		ugc	−1
*trnR*	13588–13637	50		ucg	−9
*trnN*	13629–13690	62		guu	−10
*trnS1*	13681–13737	57		acu	−1
*nad2*	13737–14660	924	GTG/TAG		1

**Table 3:** Base composition and skewness of *Aspersentis megarhynchus.*

Location/Species	A%	T%	C%	G%	AT%	AT-skew	GC-skew	Total
Whole mitochondrial genome	25.43	39.18	11.83	23.56	64.61	−0.21	0.33	14661
Protein coding genes (PCGs)	23.60	40.16	11.76	24.48	63.75	−0.26	0.35	10489
1st codon	27.04	32.92	10.72	29.32	59.96	−0.10	0.46	3499
2nd codon	15.77	48.87	13.73	21.63	64.64	−0.51	0.22	3495
3rd codon	27.98	38.68	10.84	22.49	66.67	−0.16	0.35	3495
tRNAs	30.52	39.12	9.25	21.10	69.64	−0.12	0.39	1232
rRNAs	33.20	36.35	10.84	19.61	69.54	−0.05	0.29	1494
*rrnS*	34.78	35.48	10.09	19.65	70.26	−0.01	0.32	575
*rrnL*	32.24	36.73	11.40	19.63	68.97	−0.07	0.27	912
Non-coding region 1	26.16	35.26	14.48	24.10	61.42	−0.15	0.25	967
Non-coding region 2	30.87	31.97	15.03	22.13	62.84	−0.02	0.19	366

**Figure 3:**
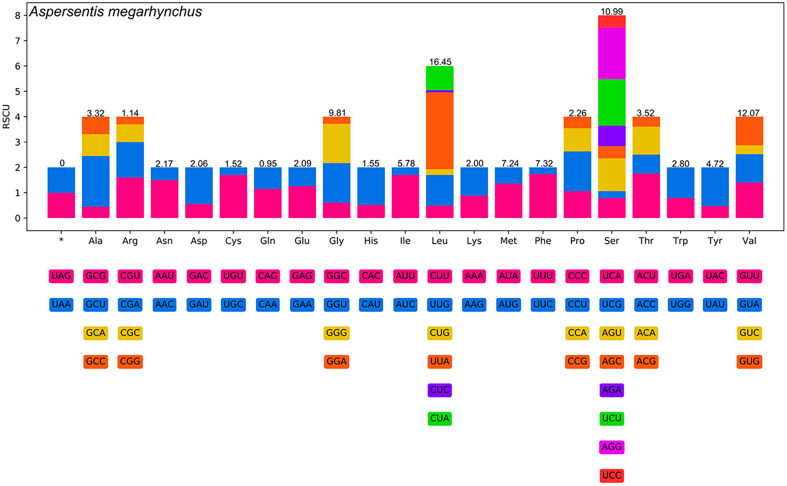
Relative synonymous codon usage (RSCU) of *Aspersentis megarhynchus*. The codon families (in alphabetical order) are labelled on the *x*-axis. Values on the top of each bar represent amino acid usage in percentage.

The 12 PCGs of the present mitogenome is 10,489 bp in length (excluding termination codons) and encoded 3,495 amino acids. The size of 12 PCGs varied from 276 bp (*nad4L*) to 1,662 bp (*nad5*) (Fig. 2, Table 2). Among the 12 PCGs of *A. megarhynchus*, 7 genes (*cox1*, *cox2*, *nad2*, *nad3*, *nad4L*, *nad5*, and *cytb*) used GTG as the start codon, while 3 genes (*nad1*, *nad6*, and *cox3*) used ATG. Meanwhile, ATA and ATT were used by the *atp6* and *nad4* genes, respectively. There are 4 genes (*cox1*, *cox3*, *cytb*, and *atp6*) that used TAA as a termination codon, and 4 genes (*nad4L*, *nad5*, *cox2*, and *nad2*) that used TAG as a termination codon. The 4 remaining genes (*nad1*, *nad3*, *nad4*, and *nad6*) were inferred to terminate with incomplete stop codon T (Table 2). In the 12 PCGs of the mitogenome of *A. megarhynchus*, TTA for leucine (8.30%) is the most frequently used codon, followed by TTT for phenylalanine (6.35%) and ATA for methionine (4.92%), ATA for isoleucine (4.92%), while CGG for arginine is the least used codons (0.09%) (Table 4). Leucine (16. 5%) is the most frequently used amino acid in the PCGs of *A. megarhynchus*, followed by valine (12.1%) and serine (11.0%) (Table 4). Detailed information on overall codon usage and RSCU for the construction of 12 PCGs is shown in Figure 3.

**Table 4:** Genetic code and codon usage for 12 PCGs in the mitochondrial genome of *Aspersentis megarhynchus*.

Codon	aa	No.	%	Codon	aa	No.	%
TAG hgbggggghg	*	0	0	CTC	Leu	9	0.26
TAA	*	0	0	CTA	Leu	91	2.60
GCG	Ala	13	0.37	AAA	Lys	31	0.89
GCT	Ala	58	1.66	AAG	Lys	39	1.12
GCA	Ala	25	0.72	ATA	Met	172	4.92
GCC	Ala	20	0.57	ATG	Met	81	2.32
CGT	Arg	16	0.46	TTT	Phe	222	6.35
CGA	Arg	14	0.40	TTC	Phe	34	0.97
CGC	Arg	7	0.20	CCC	Pro	21	0.60
CGG	Arg	3	0.09	CCT	Pro	31	0.89
AAT	Asn	57	1.63	CCA	Pro	18	0.52
AAC	Asn	19	0.54	CCG	Pro	9	0.26
GAC	Asp	20	0.57	TCA	Ser	38	1.09
GAT	Asp	52	1.49	TCG	Ser	13	0.37
TGT	Cys	45	1.29	AGT	Ser	62	1.77
TGC	Cys	8	0.23	AGC	Ser	23	0.66
CAG	Gln	19	0.54	AGA	Ser	39	1.12
CAA	Gln	14	0.40	TCT	Ser	88	2.52
GAG	Glu	46	1.32	AGG	Ser	98	2.80
GAA	Glu	27	0.77	TCC	Ser	23	0.66
GGC	Gly	52	1.49	ACT	Thr	54	1.55
GGT	Gly	134	3.83	ACC	Thr	23	0.66
GGG	Gly	133	3.81	ACA	Thr	34	0.97
GGA	Gly	24	0.69	ACG	Thr	12	0.34
CAC	His	14	0.40	TGA	Trp	39	1.12
CAT	His	40	1.14	TGG	Trp	59	1.69
ATT	Ile	172	4.92	TAC	Tyr	39	1.12
ATC	Ile	30	0.86	TAT	Tyr	126	3.61
CTT	Leu	47	1.34	GTT	Val	148	4.23
TTG	Leu	116	3.32	GTA	Val	118	3.38
CTG	Leu	22	0.63	GTC	Val	37	1.06
TTA	Leu	290	8.30	GTG	Val	119	3.40

Two ribosomal RNAs, *rrnL* and *rrnS*, are 912 bp and 575 bp in size, with 69.0% and 70.3% A + T content, respectively. The *rrnL* is located between *trnY* and *trnL1*, and *rrnS* is located between *trnM* and *trnF*. In the complete mitogenome of *A. megarhynchus*, 22 tRNAs are identified with lengths ranging from 50 bp (*trnR*) to 67 bp (*trnQ*). Among them, three tRNAs (*trnN*, *trnW,* and *trnV*) were predicted to be folded into a typical cloverleaf secondary structure. The four tRNAs (*trnR, trnC, trnQ*, and *trnS1*) lack a (DHU) arm. The remaining tRNAs lack a TψC (T) arm. The lengths of 22 tRNAs and their anticodon secondary structures are provided in Table 2 and Supplementary file 2.

**Figure 4:**
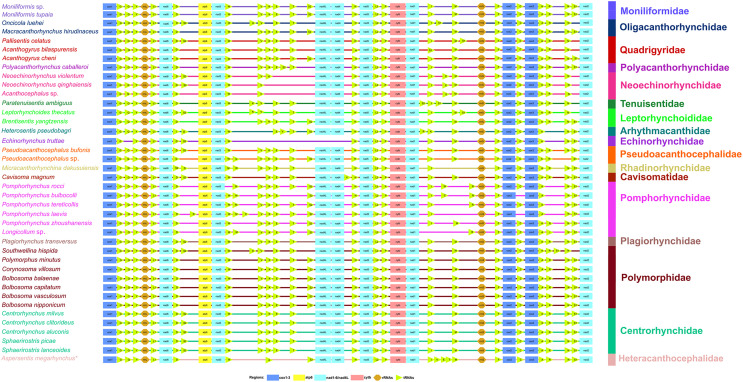
Comparison of the linearized mitochondrial genome arrangement for acanthocephalans species. All genes are transcribed in the same direction from left to right. The tRNAs are labelled by a single-letter code for the corresponding amino acid. *Aspersentis megarhynchus* is indicated using an asterisk (*).

In the mitogenome of *A. megarhynchus*, the gene arrangement of PCGs and rRNAs is in the typical order of acanthocephalans: *cox1*, *rrnL*, *nad6*, *atp6*, *nad3*, *nad4L*, *nad4*, *nad5*, *cytb*, *nad1*, *rrnS*, *cox2*, *cox3*, and *nad2*. However, the rearrangement of several tRNAs (*trnV* + *trnE + trnT* located between *trnI* and *trnM*) occurred in the mitogenome of *A. megarhynchus* (Figs. 2 and 4).

#### Phylogenetic analyses

**Figure 5:**
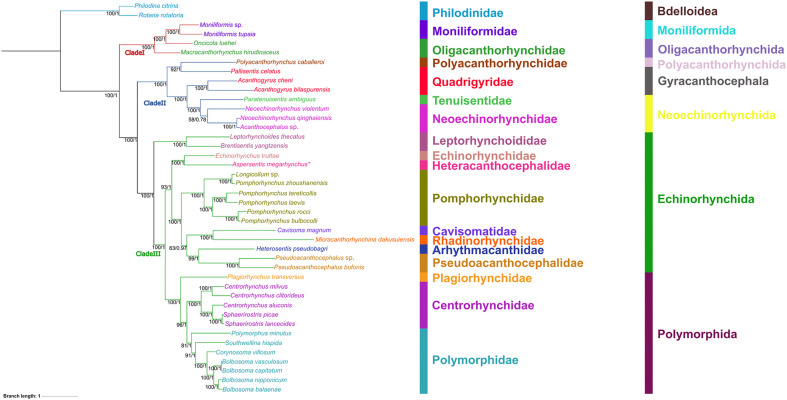
Phylogenetic analyses of Acanthocephala inferred from the ML and BI methods based on concatenating amino acid sequences of 12 protein-coding genes (PCGs) of mitochondrial genome. *Rotaria rotatoria* and *Philodina citrina* are the out-group. *Aspersentis megarhynchus* is indicated using an asterisk (*).

Phylogenetic results based on concatenated amino acid sequences of 12 protein-coding genes using the ML and BI methods have almost identical topologies, which supported the division of the phylum Acanthocephala into three large monophyletic clades (clades I, II, and III) (Fig. 5). Clade I consists of *Macracanthorhynchus hirudinaceus* (Pallas, 1781), *Oncicola luehei* (Travassos, 1917), *Moniliformis tupaia* Chen, Yu, Ma, Zhao, Cao & Li, 2024, and *Moniliformis* sp., representing the class Archiacanthocephala. Clade II includes the representatives of Gyracanthocephala and Neoechinorhynchida, representing the class Eoacanthocephala (*Polyacanthorhynchus caballeroi* Diaz-Ungria et Rodrigo, 1960 belonging to the class Polyacanthocephala nested into representatives of Eoacanthocephala). Clade III contains species of Echinorhynchida and Polymorphida, representing the class Palaeacanthocephala. *Aspersentis megarhynchus* belonging to the family Heteracanthocephalidae displayed a sister relationship with *Echinorhynchus truttae* Schrank, 1788 belonging to the family Echinorhynchidae in the order Echinorhynchida.

## Discussion

In the order Echinorhynchida, only 14 acanthocephalan species belonging to seven different families have been sequenced for the mitogenomes [11, 41, 57, 58, 60, 63], and over 45% of families in Echinorhynchida still have all representatives with unknown mitogenomic data. In the present study, the complete mitochondrial genome of *A. megarhynchus* was provided for the first time, which represented the first mitogenomic data for the genus *Aspersentis* and also for the family Heteracanthocephalidae.

In Echinorhynchida, the size of the mitogenome of *A. megarhynchus* (14,661 bp) is similar to that of two pomphorhynchid species *Pomphorhynchus zhoushanensis* Li, Chen, Amin & Yang, 2017 and *Longicollum* sp. (both 14,632 bp), but the overall A + T content in the mitogenome of *A. megarhynchus* (64.6%) is higher than that of *P. zhoushanensis* and *Longicollum* sp. (both 55.8%), and is similar to that of the echinorhynchid species *Echinorhynchus truttae* (63.1%) [60]. Previous studies proved that the organization and arrangements of the 12 PCGs and 2 rRNAs in the phylum Acanthocephala are conserved, whereas the position of tRNAs tends to be highly variable among different families or genera [9, 10, 16, 18, 63]. Comparative mitochondrial genomic analysis revealed that several tRNA gene (*trnV*, *trnE* and *trnT*) rearrangement events occurred in the mitogenomes of *A. megarhynchus*. The tRNA gene arrangement in the mitogenome of *A. megarhynchus* is different from all of the known mitogenomes of acanthocephalans.

Amin *et al.* [4] sequenced the 18S data of *A. megarhynchus* and constructed the phylogenetic tree of Echinorhynchida based on the 18S sequences using the ML and BI methods, whose results supported the validity of the Heteracanthocephalidae, but did not solve the phylogenetic relationships between the Heteracanthocephalidae and some other families in Echinorhynchida (i.e., Echinorhynchidae, Pomphorhynchidae, Paracanthocephalidae, Rhadinorhynchidae, and Arhythmacanthidae). The present mitogenomic phylogeny also indicated that Heteracanthocephalidae represented a separate family, which was consistent with the phylogenetic results based only on 18S data. Moreover, the present study represented the first attempt to investigate the systematic position of the Heteracanthocephalidae in the order Echinorhynchida using phylogenetic analyses based on mitogenomic data. The phylogenetic results based on concatenating the amino acid sequences of 12 PCGs strongly suggested a close affinity between the families Heteracanthocephalidae and Echinorhynchidae in the order Echinorhynchida, which rejected the previous proposals by Van Cleave [59] and Petrochenko [49]. The present study enriched the resource of genetic data and contributed to revealing the patterns of mitogenomic evolution of the Heteracanthocephalidae, and represented a substantial step towards clarifying the phylogenetic relationships of different families in Echinorhynchida.

## Figures and Tables

**Supplementary file 2:**
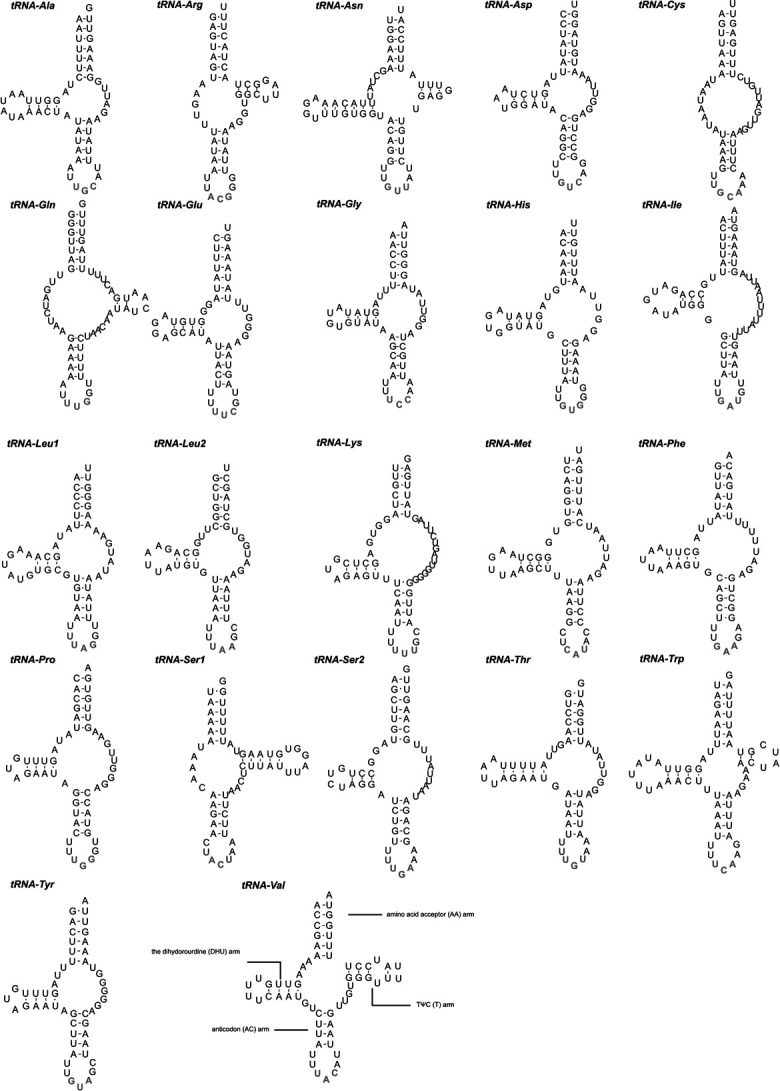
The predicted secondary structures of 22 tRNAs in the mitogenome of *Aspersentis megarhynchus* (Watson-Crick bonds indicated by lines, GU bonds indicated by dots, red bases representing anticodons). The tRNAs are labelled with the abbreviations of their corresponding amino acids according to the IUPAC-IUB code.

## Supplementary information

The supplementary material of this article is available at https://doi.org/www.parasite-journal.org/10.1051/parasite/2024064/olm. *Supplementary file 1*: Python script used for calculating relative synonymous codon usage (RSCU).
