# Supplementary material for: Novel gene arrangement in the mitochondrial genome of Aspersentis megarhynchus (Acanthocephala, Echinorhynchida, Heteracanthocephalidae), and its phylogenetic implications
Source: Parasite. 2024 Oct 8;31:63. doi: 10.1051/parasite/2024064 (PMC11460826; doi:10.1051/parasite/2024064)
Supplement: Supplementary file 1 — Python script used for calculating relative synonymous codon usage (RSCU). [file parasite-31-63-s1.pdf]

```
From collections import Counter
import matplotlib.pyplot as plt
from pandas import DataFrame
from Bio import SeqIO
import pandas as pd
import numpy as np
import argparse

table5 = {    "TTT": "Phe", "TTC": "Phe", "TTA": "Leu", "TTG": "Leu",
              "TCT": "Ser", "TCC": "Ser", "TCA": "Ser", "TCG": "Ser",
              "TAT": "Tyr", "TAC": "Tyr", "TAA": " * ", "TAG": " * ",
              "TGT": "Cys", "TGC": "Cys", "TGA": "Trp", "TGG": "Trp",
              "CTT": "Leu", "CTC": "Leu", "CTA": "Leu", "CTG": "Leu",
              "CCT": "Pro", "CCC": "Pro", "CCA": "Pro", "CCG": "Pro",
              "CAT": "His", "CAC": "His", "CAA": "Gln", "CAG": "Gln",
              "CGT": "Arg", "CGC": "Arg", "CGA": "Arg", "CGG": "Arg",
              "ATT": "Ile", "ATC": "Ile", "ATA": "Met", "ATG": "Met",
              "ACT": "Thr", "ACC": "Thr", "ACA": "Thr", "ACG": "Thr",
              "AAT": "Asn", "AAC": "Asn", "AAA": "Lys", "AAG": "Lys",
              "AGT": "Ser", "AGC": "Ser", "AGA": "Ser", "AGG": "Ser",
              "GTT": "Val", "GTC": "Val", "GTA": "Val", "GTG": "Val",
              "GCT": "Ala", "GCC": "Ala", "GCA": "Ala", "GCG": "Ala",
              "GAT": "Asp", "GAC": "Asp", "GAA": "Glu", "GAG": "Glu",
              "GGT": "Gly", "GGC": "Gly", "GGA": "Gly", "GGG": "Gly", }

##RSCU_count
def get_synonymous_codons(genetic_code_dict):
    # invert the genetic code dictionary to map each amino acid to its codons
    codons_for_amino_acid = {}
    for codon, amino_acid in list(genetic_code_dict.items()):
        codons_for_amino_acid[amino_acid] = codons_for_amino_acid.get(amino_acid, [])
        codons_for_amino_acid[amino_acid].append(codon)
        #print(genetic_code_dict.items())
    # create dictionary of synonymous codons
    # Example: {'CTT': ['CTT', 'CTG', 'CTA', 'CTC', 'TTA', 'TTG'], 'ATG': ['ATG']...}
    return {codon: codons_for_amino_acid[genetic_code_dict[codon]] for codon in list(genetic_code_dict.keys())}


def RSCU(sequences, genetic_code_dict=table5):
    if not isinstance(sequences, (list, tuple)):
        raise ValueError("Be sure to pass a list of sequences, not a single sequence. To find the RSCU of a single sequence, pass it as a one element list.")
```

```
    # ensure all input sequences are divisible by three
    for sequence in sequences:
        if len(sequence) % 3 != 0:
            raise ValueError("Input sequence not divisible by three")
        if not sequence:
            raise ValueError("Input sequence cannot be empty")

    # count the number of each codon in the sequences
    #sequences =['ACTGTCGTA','TTAATGCTGAGT']
    sequences = [[sequence[i:i + 3].upper() for i in range(0, len(sequence), 3)] for sequence in
sequences]
    # flat list of all codons (to be used for counting)
    codons = []
    for x in sequences:
        codons.extend(x)
    # The number of occurrences of each codon was calculated
    counts = Counter(codons)

    # "if a certain codon is never used in the reference set... assign [its
    # count] a value of 0.5" (page 1285)

    for codon in table5:
        if counts[codon] == 0:
            counts[codon] = 0.5

    # determine the synonymous codons for the genetic code
    synonymous_codons = get_synonymous_codons(genetic_code_dict)

    # hold the result as it is being calulated
    result = {}
    # calculate RSCU values
    for codon in genetic_code_dict:
        result[codon] = counts[codon] / ((len(synonymous_codons[codon]) ** -1) *
(sum((counts[_codon] for _codon in synonymous_codons[codon]))))
        #print(counts[codon])
        #print(len(synonymous_codons[codon]))
        #print(sum((counts[_codon] for _codon in synonymous_codons[codon])))

    sum_codons={}
    for codon in table5:
        sum_codons[codon]=[counts[codon],result[codon]]

    return sum_codons
```

```
def get_seq3(remainder, sequence, header):
    r = remainder
    line_new = sequence[:-r]
    stop_codon = sequence[-r:]
    len_seq2 = len(line_new)
    remainder2 = len_seq2 % 3
    print(header + ' ' + "incomplete stop codon" + ':' + stop_codon + ';' + str(r) + '>>' +
str(remainder2))
    return line_new


def read_fas_out_mul3(input):
    name = input.split(".")[0]
    ofile = name + '_mul3.fas'
    outputfile = str(ofile)
    # print(outputfile)
    out_fas = open(outputfile, 'a')
    with open(input) as input_f:
        #fasta = {}
        for line in input_f:
            line = line.strip()
            if line[0] == '>':
                id = line[1:]
                id2 = id.split(';')
                header = id2[1]
            else:
                sequencelist=[]
                sequencelist.append(line)
                sequence = line
                len_seq = len(sequence)
                #enter the sequence name
                out_fas.write('>' + header + '\n')
                #calculates whether the sequence is a multiple of 3
                remainder = len_seq % 3
                if remainder == 0:
                    seq = sequence
                else:
                    seq = get_seq3(remainder, sequence, header)
                out_fas.write(seq + '\n')
    input_f.close()
    out_fas.close()
    return outputfile
```

```
##plot_RSCU_bar
def make_stacked_plot(df_color):
    # color
    color = {1: '#FFFFAA', 1.5: '#FF9D6F', 2: '#0080FF', 2.5: '#FF2D2D', 3: '#E800E8', 3.5: '#00DB00', 4: '#8600FF',
             4.5: '#FF5809', 5: '#EAC100', 5.5: '#0072E3', 6: '#FF0080'}
    # canvas
    plt.figure(figsize=(15, 10))
    # quadrant 1
    plt.subplot(211)
    # rscu table perspective
    dfrscu = df_color.pivot_table(index="AA", columns='color', values='RSCU', aggfunc=sum, fill_value=0)
    #sets the bottom value of the stack plot and creates a list of color values
    margin_bottom = np.zeros(len(df_color['AA'].drop_duplicates()))
    heights_list = df_color['color'].drop_duplicates()
    # stacking
    for num, h in enumerate(heights_list):
        values = list(dfrscu[h])
        plt.bar(x=dfrscu.index, height=dfrscu[h].fillna(0), bottom=margin_bottom, facecolor=color[h], label=h, alpha=1)
        margin_bottom += values
    # Y-axis heading
    plt.ylabel("RSCU")
    # set the xy axis range
    plt.ylim((-0.5, max(margin_bottom) + 0.5))
    plt.xlim((-1, len(dfrscu.index)))

    # quadrant 2
    plt.style.use('ggplot')
    ax2 = plt.subplot(212)
    # set the 2nd quadrant background blank
    ax2.patch.set_facecolor('none')
    # codnon table perspective
    dfcodon = df_color.pivot_table(index="AA", columns='color', values='codon', aggfunc=sum, fill_value='')
    # xy location with codon content
    xs = list(range(0, len(dfcodon.index.values)))
    aas = dfcodon.index.values
    ys = dfcodon.columns.values
    # set the xy axis range and remove the xy axis
    plt.ylim((min(dfcodon.columns.values) - 0.5, max(dfcodon.columns.values)))
    plt.xlim((-1, len(dfcodon.index)))
```

```
    plt.xticks([])
    plt.yticks([])
    # codon mapping diagram
    for x in xs:
        for y in ys:
            ax2.text(x, y, dfcodon.iloc[x][y], va="center", ha="center",
                     bbox=dict(boxstyle="round", facecolor=color[y], alpha=1))
    # save picture
    plt.savefig(fname="RSCU.svg", format="svg")
    plt.savefig(fname="RSCU.png", format="png", dpi=300)
    plt.show()


def RSCU_process(input_fasta):
    mul3_seq = read_fas_out_mul3(input_fasta)
    abb = (input_fasta).split(".")[0]
    seqs = [rec.seq for rec in SeqIO.parse(mul3_seq, 'fasta')]
    rscu = RSCU(seqs, table5)

    # statistical result
    df_rscu_sum = pd.DataFrame(columns=['AA', 'codon', 'count_num', 'rscu_val'])
    for codon in rscu.keys():
        info_list = [table5[codon], codon, rscu[codon][0], round(rscu[codon][1], 3)]
        df_rscu_sum.loc[codon] = info_list

    df_rscu_sum.to_excel('{}_sum.xlsx'.format(abb))
    # draw a picture
    df_rscu_color = pd.DataFrame(columns=['AA', 'codon', 'RSCU', 'color'])
    color = {}
    for codon in rscu.keys():
        if table5[codon] not in color:
            color[table5[codon]] = 6
        else:
            color[table5[codon]] -= 0.5
        info_list = [table5[codon], codon, round(rscu[codon][1], 3), color[table5[codon]]]
        df_rscu_color.loc[codon] = info_list
    df_rscu_color['codon'] = df_rscu_color['codon'].str.replace('T', 'U')
    make_stacked_plot(df_rscu_color)
    df_rscu_color.to_excel('{}_color.xlsx'.format(abb))

##base_count_ratio
def count_seq(sequence):
    codon1 = []
    codon2 = []
```

```
codon3 = []

total = len(sequence)
sequence = [sequence[i:i + 3].upper() for i in range(0, len(sequence), 3)]
for codon in sequence:
    codon1.append(codon[0])
    try:
        codon2.append(codon[1])
    except:
        continue
    try:
        codon3.append(codon[2])
    except:
        continue

count_codon1=Counter(codon1)
count_codon2=Counter(codon2)
count_codon3=Counter(codon3)

seqA=count_codon1['A']+count_codon2['A']+count_codon3['A']
seqT=count_codon1['T']+count_codon2['T']+count_codon3['T']
seqC=count_codon1['C']+count_codon2['C']+count_codon3['C']
seqG=count_codon1['G']+count_codon2['G']+count_codon3['G']
#total=seqA+seqT+seqC+seqG

#dict_seq={'A':seqA, 'T':seqT, 'C':seqC, 'G':seqG}
'''
dict_c1=count_codon1
dict_c2=count_codon2
dict_c3=count_codon3
'''

#codon1 的 ATCG

cod1A=count_codon1['A']
cod1T=count_codon1['T']
cod1C=count_codon1['C']
cod1G=count_codon1['G']
post_1 = len(codon1)
#post_1 = cod1A + cod1T + cod1C + cod1G
#dict_c1 = {'A': codon1A, 'T': codon1T, 'C': codon1C, 'G': codon1G}

#codon2 的 ATCG

cod2A=count_codon2['A']
cod2T=count_codon2['T']
```

```
    cod2C=count_codon2['C']
    cod2G=count_codon2['G']
    post_2 = len(codon2)
    #post_2 = cod2A + cod2T + cod2C + cod2G
    #dict_c2 = {'A': codon2A, 'T': codon2T, 'C': codon2C, 'G': codon2G}

    #codon3 的 ATCG

    cod3A=count_codon3['A']
    cod3T=count_codon3['T']
    cod3C=count_codon3['C']
    cod3G=count_codon3['G']
    post_3 = len(codon3)
    #post_3 = cod3A + cod3T + cod3C + cod3G
    #dict_c3 = {'A': codon3A, 'T': codon3T, 'C': codon3C, 'G': codon3G}

    #[dict_seq,dict_c1,dict_c2,dict_c3]
    return [seqA,seqT,seqC,seqG,total,
            cod1A,cod1T,cod1C,cod1G,post_1,
            cod2A,cod2T,cod2C,cod2G,post_2,
            cod3A,cod3T,cod3C,cod3G,post_3]

def get_per_tab(input_table):
    pertable=input_table.copy()
    #print(input_table)

    # pertable.insert(1,column='A%',value=(sum_table['A']/sum_table['Total'])*100)
    pertable["A%"] = (pertable['A'] / pertable['Total']) * 100
    pertable["T%"] = (pertable['T'] / pertable['Total']) * 100
    pertable["C%"] = (pertable['C'] / pertable['Total']) * 100
    pertable["G%"] = (pertable['G'] / pertable['Total']) * 100
    pertable["AT%"] = ((pertable['A']+pertable['T'])/ pertable['Total']) * 100
    # 'A-1', 'T-1', 'C-1', 'G-1', 'Post-1',
    pertable["A-1%"] = (pertable['A-1'] / pertable['Post-1']) * 100
    pertable["T-1%"] = (pertable['T-1'] / pertable['Post-1']) * 100
    pertable["C-1%"] = (pertable['C-1'] / pertable['Post-1']) * 100
    pertable["G-1%"] = (pertable['G-1'] / pertable['Post-1']) * 100
    pertable["AT-1%"] = ((pertable['A-1'] + pertable['T-1']) / pertable['Post-1']) * 100

    pertable["A-2%"] = (pertable['A-2'] / pertable['Post-2']) * 100
    pertable["T-2%"] = (pertable['T-2'] / pertable['Post-2']) * 100
    pertable["C-2%"] = (pertable['C-2'] / pertable['Post-2']) * 100
    pertable["G-2%"] = (pertable['G-2'] / pertable['Post-2']) * 100
    pertable["AT-2%"] = ((pertable['A-2'] + pertable['T-2']) / pertable['Post-2']) * 100
```

```
    pertable["A-3%"] = (pertable['A-3'] / pertable['Post-3']) * 100
    pertable["T-3%"] = (pertable['T-3'] / pertable['Post-3']) * 100
    pertable["C-3%"] = (pertable['C-3'] / pertable['Post-3']) * 100
    pertable["G-3%"] = (pertable['G-3'] / pertable['Post-3']) * 100
    pertable["AT-3%"] = ((pertable['A-3'] + pertable['T-3']) / pertable['Post-3']) * 100

    return pertable

def get_sum_table(input_table):
    sumtable=input_table.copy()
    forward_strand_table = sumtable[(sumtable['strand'] == '(+)')]
    reverse_strand_table = sumtable[(sumtable['strand']=='(-)')]
    sumtable.loc['totoal_sum'] = sumtable[['A', 'T', 'C', 'G', 'Total', 'A-1', 'T-1', 'C-1', 'G-1',
                                  'Post-1', 'A-2', 'T-2', 'C-2', 'G-2', 'Post-2', 'A-3', 'T-3', 'C-3',
                                  'G-3', 'Post-3']].apply(lambda x: x.sum())
    #+
    if len(sumtable[(sumtable['strand']=='(+)')]) > 0 :
        sumtable.loc['forward_strand'] = forward_strand_table[['A', 'T', 'C', 'G', 'Total', 'A-1',
'T-1', 'C-1', 'G-1',
                                      'Post-1', 'A-2', 'T-2', 'C-2', 'G-2', 'Post-2', 'A-3', 'T-3', 'C-3',
                                      'G-3', 'Post-3']].apply(lambda x: x.sum()).copy()
    else:
        print("no (+) strand gene")
    #-
    if len(sumtable[(sumtable['strand']=='(-)')]) > 0 :
        sumtable.loc['reverse_strand'] = reverse_strand_table[['A', 'T', 'C', 'G', 'Total', 'A-1', 'T-1',
'C-1', 'G-1',
                                      'Post-1', 'A-2', 'T-2', 'C-2', 'G-2', 'Post-2', 'A-3', 'T-3', 'C-3',
                                      'G-3', 'Post-3']].apply(lambda x: x.sum()).copy()
    else:
        print("no (-) strand gene")
    final_table=get_per_tab(sumtable)
    final_table[['A%', 'T%', 'C%', 'G%', 'AT%', 'A-1%', 'T-1%', 'C-1%','G-1%', 'AT-1%', 'A-2%',
'T-2%', 'C-2%',
                 'G-2%', 'AT-2%', 'A-3%','T-3%', 'C-3%', 'G-3%', 'AT-3%']]=final_table[['A%',
'T%', 'C%', 'G%',
                  'AT%', 'A-1%', 'T-1%', 'C-1%','G-1%', 'AT-1%', 'A-2%', 'T-2%', 'C-2%',
'G-2%', 'AT-2%', 'A-3%',
                  'T-3%', 'C-3%', 'G-3%', 'AT-3%']].astype('float').round(2)
    final_table[['A', 'T', 'C', 'G', 'Total', 'A-1', 'T-1', 'C-1', 'G-1','Post-1', 'A-2', 'T-2', 'C-2',
                 'G-2', 'Post-2', 'A-3', 'T-3', 'C-3','G-3', 'Post-3']] = final_table[['A', 'T', 'C',
                'G', 'Total', 'A-1', 'T-1', 'C-1', 'G-1','Post-1', 'A-2', 'T-2', 'C-2', 'G-2', 'Post-2',
                'A-3', 'T-3', 'C-3','G-3', 'Post-3']].astype('int')
    return final_table
```

```
def get_seqs_count(input_fasta):
    base_count_dftmp=DataFrame(
            columns=['strand',
                     'A', 'T', 'C', 'G', 'Total',
                     'A-1', 'T-1', 'C-1', 'G-1', 'Post-1',
                     'A-2', 'T-2', 'C-2', 'G-2', 'Post-2',
                     'A-3', 'T-3', 'C-3', 'G-3', 'Post-3'])
    #count—seq
    for rec in SeqIO.parse(input_fasta, 'fasta'):
        sequence=rec.seq
        id=(rec.id).split(';')[1]
        #print(id)
        strand=(rec.id).split(']')[1]
        base_count_list=count_seq(sequence)
        base_count_list.insert(0, strand)
        base_count_dftmp.loc[id] = base_count_list
    #sum_table.to_excel("{0}_{1}__{2}.xlsx".format(prefix, line, data))
    count_table=base_count_dftmp.sort_values(by=['strand'], ascending=False)
    final_sum_table=get_sum_table(count_table)
    abb = str(input_fasta)
    final_sum_table.to_excel('{}_base_count.xlsx'.format(abb))


if __name__ == "__main__":
    parser = argparse.ArgumentParser()
    parser.add_argument('--input', '-i',
                        type=str,
                        help='input file in fasta format')
    args = parser.parse_args()


    RSCU_process(args.input)
    get_seqs_count(args.input)
    print("Be careful! If the total length is not equal to the sum of ATCG,"
          " ambiguous bases exist in the sequence , eg. Y K M ")
```
